# Plantar sensory stimulation and its impact on gait and lower limb motor function in individuals with stroke: A systematic review and meta-analysis

**DOI:** 10.1371/journal.pone.0315097

**Published:** 2024-12-06

**Authors:** Adamu Adamu Ahmad, Duangporn Suriyaamarit, Akkradate Siriphorn

**Affiliations:** 1 Human Movement Performance Enhancement Research Unit, Department of Physical Therapy, Faculty of Allied Health Sciences, Chulalongkorn University, Bangkok, Thailand; 2 Department of Physiotherapy, Aminu Kano Teaching Hospital, Kano, Nigeria; Opole University of Technology: Politechnika Opolska, POLAND

## Abstract

**Background:**

Stroke frequently leads to motor impairments, with almost half of the affected individuals experiencing diminished sensation, impacting their overall quality of life and autonomy. Rehabilitation efforts, however, often overlook somatosensory functions of the lower limbs. While plantar sensory stimulation activates receptors in the foot sole, its precise impact on the motor functions and gait of individuals with stroke is yet to be ascertained.

**Objectives:**

This systematic review and meta-analysis aimed to examine the effects of sensory interventions on gait and lower limb motor function in individuals with stroke.

**Methods:**

We searched eight databases from inception to December 2023 for randomized controlled trials that investigated sensory interventions targeting gait or lower limb motor function in stroke patients. The primary outcomes included changes in gait and motor function, reported as standardized mean differences (SMD) and assessed heterogeneity (I^2^).

**Results:**

A total of [number] studies were included, covering different sensory modalities such as textured insoles, plantar vibration, and cognitive sensorimotor exercises. The interventions showed varying effectiveness, with plantar vibration therapy exhibiting a large effect size (SMD = 2.03 [1.13, 2.94]) for improving lower limb motor function, while textured insoles showed moderate effectiveness (SMD = 0.58 [0.24, 0.92]) with no heterogeneity (I^2^ = 0%). For gait, significant enhancement was seen with plantar vibration (SMD = 3.17 [2.05, 4.29]) and cognitive sensorimotor training (SMD = 2.85 [1.69, 4.02]). However, overall heterogeneity was moderate to high (I^2^ = 65% for motor function, 85% for gait), indicating variability across different studies and intervention types.

**Conclusion:**

The findings of this review and meta-analysis suggest that plantar somatosensory stimulation has the potential to improve lower limb motor function and gait in people with stroke. However, to firmly establish its efficacy as a rehabilitative tool, larger-scale and high-quality studies are requisite.

## Introduction

Stroke stands as a leading cause of severe and enduring disability, imposing significant healthcare expenditures and projecting future health economic burdens [1]. The risk of stroke escalates, particularly among individuals aged above 65 years, underscoring the pressing need for effective rehabilitation strategies to mitigate its impact on an aging population [2]. The ability to walk represents a fundamental marker of functional independence and engagement in social activities for stroke patients [3, 4]. However, following hospital discharge, a substantial proportion of stroke survivors—approximately 45%—encounter challenges in walking, characterized by an inability to ambulate or the manifestation of abnormal gait patterns with spatial and temporal asymmetries. This gait disability significantly impairs daily living activities, diminishes the overall quality of life, and may lead to secondary complications such as muscle shortening and joint impairments [5]. Consequently, timely and effective rehabilitation management is essential to enhance the quality of life for stroke patients. Even six months after experiencing a stroke, about 30% of survivors still require varying degrees of walking assistance, emphasizing the necessity for comprehensive and individualized interventions [6, 7].

Despite significant progress in identifying and treating post-stroke disability, the existing evidence is derived from heterogeneous studies, making it challenging to draw definitive conclusions regarding optimal interventions for individual patients [8]. One notable consequence of stroke is somatosensory impairment of the lower extremity, which affects a substantial proportion of stroke survivors, ranging between 45% and 56%, leading to difficulties in performing routine tasks [9, 10].

Sensory perception represents a fundamental modality for processing and interacting with the external environment [11, 12]. It enables the detection and discrimination of objects and textures, spatial awareness of body position (proprioception), and precise perception and differentiation of pain, temperature, pressure, and vibration sensations [13, 14]. Indeed, sensory perception is an essential element in human functioning, playing a pivotal role in facilitating normal physiological processes and influencing motor behaviors [13]. Notably, somatosensory input assumes particular significance in enabling accurate and adaptable motor control and in facilitating the acquisition of motor skills. This highlights the potential significance of sensory stimuli as a valuable constituent for enhancing motor rehabilitation strategies [15–17].

Recent advancements in neurorehabilitation have emphasized the critical role of somatosensory inputs in driving motor recovery through mechanisms such as neuroplasticity [18], proprioceptive feedback [19], and adaptive motor learning [19]. Sensory stimulation, particularly when applied to the plantar surface, activates mechanoreceptors and proprioceptive pathways, facilitating improved motor control and gait adaptations [20, 21]. These interventions are believed to stimulate brain regions involved in sensorimotor integration, thereby enhancing neuroplastic changes that support motor function recovery in stroke patients [22]. Technologies such as wearable sensors and robotic devices have further enhanced the precision and adaptability of these interventions, allowing for real-time feedback and individualized therapy adjustments [23]. For example, wearable devices can monitor gait parameters, providing immediate feedback to patients, while robotic devices can deliver consistent, controlled stimulation tailored to individual needs [24]. These technological innovations hold promise for improving the effectiveness of sensory-based rehabilitation strategies.

Despite the importance of somatosensory function in the lower limb, there is a lack of emphasis on rehabilitation interventions targeting this aspect. One such intervention, plantar sensory stimulation, involves activating the sensory receptors on the sole of the foot. However, the precise impact of plantar sensory stimulation on lower limb motor function and gait in stroke patients has not been clearly elucidated. Thus, the objective of this study was to conduct a systematic review and meta-analysis to investigate the effects of plantar sensory stimulation in improving lower limb motor function and gait in people with stroke.

## Materials and methods

This study employs a rigorous and systematic methodology, consisting of a systematic review and meta-analysis, with strict adherence to the guidelines provided by the Preferred Reporting Items for Systematic Reviews and Meta-Analyses (PRISMA) framework [25]. To ensure utmost transparency and credibility, the protocol for this study has been officially registered at the International Prospective Register of Systematic Reviews (PROSPERO) with the registration number CRD42021290399 on December 21, 2021.

### Inclusion and exclusion criteria

To guarantee the reliability and precision of our findings, the selected studies underwent an exhaustive screening process. We only considered randomized controlled trials involving adult patients aged above 18 years, diagnosed with stroke, and exhibiting motor impairments. Furthermore, to maintain consistency and coherence, the selected studies were required to have employed well-established assessments of lower limb motor function and gait in stroke patients. We also took into account studies investigating the effects of plantar sensory stimulation, either as a standalone intervention or when used in conjunction with other treatment regimens, thereby encompassing a comprehensive evaluation of its efficacy. In this review, we specifically considered research articles that were published in English. On the other hand, we deliberately excluded studies that solely focused on examining upper limb function in stroke patients. Additionally, studies involving participants under 18 years of age, those with bilateral stroke, or other neurological ailments were excluded.

### Data sources search terms and search strategy

Our systematic review process rigorously adhered to the PRISMA guidelines, reflecting our commitment to conducting a comprehensive search for relevant literature. To ensure inclusivity, we conducted an extensive search of studies published from 1978 to September 2023, utilizing reputable and scholarly databases, specifically PubMed, Scopus, Web of Science, and Google Scholar (S1 Table). To enhance the reliability of our findings, two independent reviewers (AA and DS) diligently implemented the electronic search strategy within these databases, up to September 2023. Our search strategy employed relevant and precise terms related to stroke, plantar stimulation, lower limb motor function, and gait, thus maximizing the retrieval of pertinent studies. We judiciously combined free-text words and Medical Subject Headings (MeSH) terms using appropriate Boolean logic operators (AND and OR) to optimize the search results. Moreover, we adopted the Population, Intervention, Comparator, and Outcome (PICO) framework as the foundation of our research question, ensuring a focused and structured approach to our investigation.

Participants: Our study comprised a representative population of adults who had experienced their first stroke, diagnosed by qualified specialists, and were currently undergoing hospital-based inpatient or outpatient rehabilitation. We intentionally imposed no restrictions on participants’ ethnicity, gender, or other demographic features to ensure inclusivity and a more accurate reflection of the population under investigation.

Interventions: Our investigation meticulously considered studies that involved the application of plantar sensory stimulation on stroke patients, ascertaining its effects on lower limb motor function and gait. This approach allowed for a comprehensive assessment of the potential benefits of this intervention in a well-defined context, enhancing the robustness of our conclusions.

Outcome measures: To ensure a comprehensive evaluation of lower limb recovery, we thoroughly examined all clinical measures used to assess this aspect, encompassing assessments at two distinct time points (e.g., pre- to post-intervention). Our primary focus centered on examining gait and lower limb motor function, employing widely recognized and validated tools such as the Fugl-Meyer Assessment and the Rivermead Mobility Index, thereby ensuring the accuracy and reliability of our outcomes.

Control or comparator: We meticulously considered and analyzed different types of control interventions, such as no treatment, other active interventions, usual care, and sham interventions.

### Study selection

**Fig 1:**
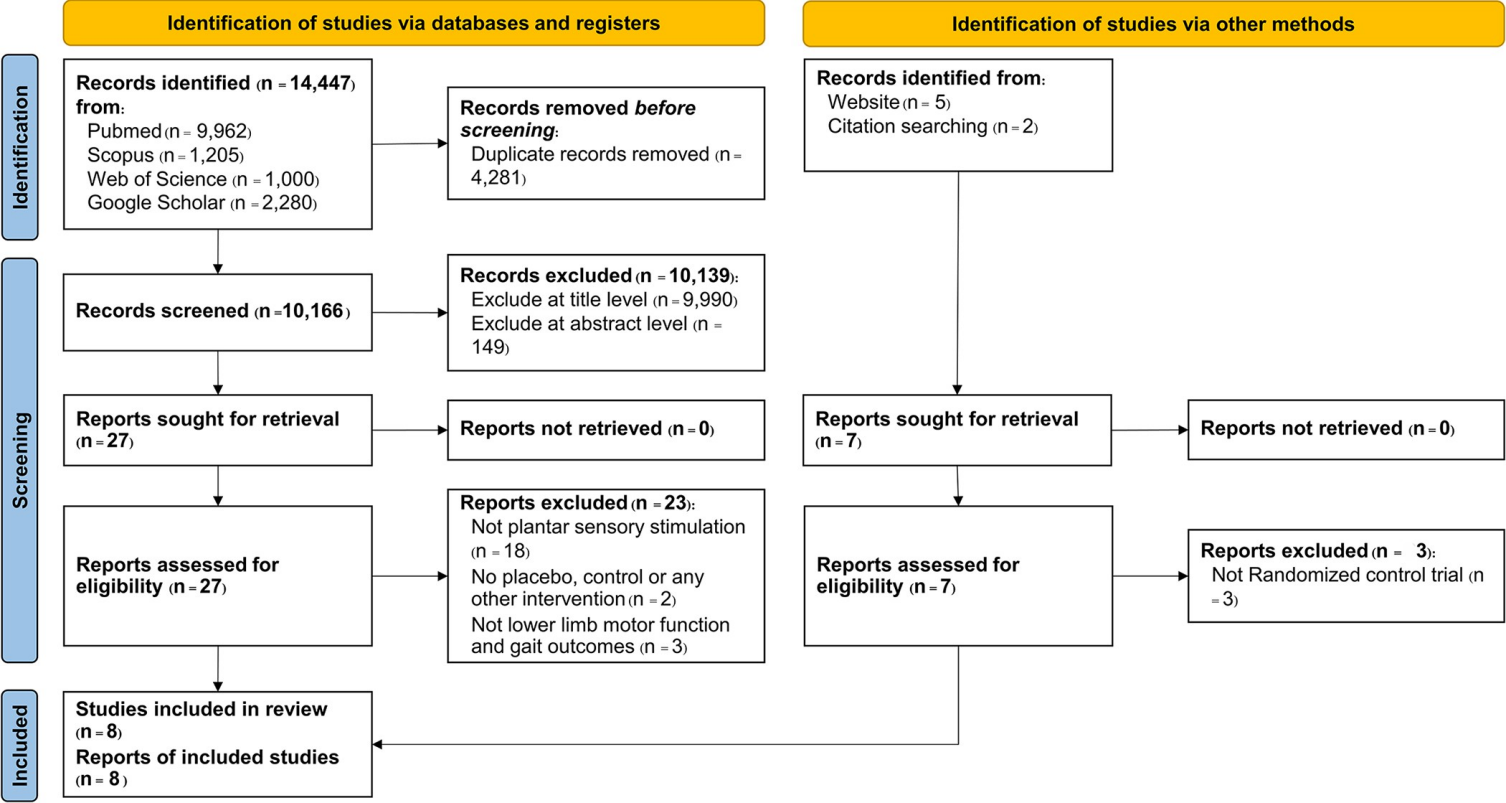
Flow of articles throughout the selection process.

All identified articles from the search strategy were exported to the CADIMA website, an open-access online tool established by Julius Kühn-Institut (JKI) for facilitating and documenting systematic reviews and further literature reviews (available at www.cadima.info). The CADIMA website automated the duplicate removal process and ensured an automated allocation of records during the screening process, considering the potential for independent and parallel assessment. Two independent reviewers, AA and DS, then screened the articles based on titles and abstracts, excluding studies that did not meet the eligibility criteria. Full texts from the selected studies were retrieved and thoroughly reviewed by both reviewers to determine their appropriateness for data extraction. In cases of disagreement regarding the eligibility of a study, AA and DS resolved the issue through a thorough review of the study criteria and discussion. Any remaining disagreements were resolved through consultation with the third reviewer, AS. Fig 1 presents the PRISMA flow chart, illustrating the search process of the study.

### Data collection/extraction process

To ensure a comprehensive and unbiased approach, we independently verified the titles and abstracts of the selected studies. Through consensus and careful examination, any duplicate entries were diligently removed. The assessment of articles was executed with precision, adhering to the predetermined selection criteria and utilizing a well-structured data extraction form. Reviewers completed these forms independently, ensuring that all relevant information, including details on participants, interventions, controls, outcomes, methodologies, study designs, follow-up/retention strategies, and any reported adverse events, was accurately captured.

### Data extraction

To ensure a thorough and unbiased approach, we independently verified the titles and abstracts of the selected studies. Through consensus and careful examination, any duplicate entries were diligently removed. The assessment of articles was executed with precision, following the predetermined selection criteria and utilizing a well-structured data extraction form. Reviewers completed these forms independently, ensuring all relevant information was captured, including details on participants, interventions, controls, outcomes, methodologies, study designs, follow-up/retention strategies, and any reported adverse events.

### Data analysis

To assess the risk of bias in the studies we selected, we utilized the revised Cochrane risk of bias tool for randomized trials (RoB 2.0) [26]. This comprehensive tool facilitated a thorough evaluation of bias in five critical domains, which include bias arising from the randomization process, bias due to deviations from intended interventions, bias related to missing outcome data, bias in the measurement of outcomes, and bias in the selection of reported results. The data from the identified studies were analyzed using Review Manager software version 5.3. To determine the effect of plantar sensory stimulation compared to control conditions, we conducted random-effects meta-analyses on the reported outcome measures for gait and lower limb motor functions. Random-effects meta-analyses were chosen as the outcome measures estimating treatment effects varied across the studies. The effect size was calculated using the Standardized Mean Difference (SMD) due to the use of diverse outcome measures. SMD was calculated by the difference in mean outcome between groups divided by the standard deviation of outcomes [27]. For statistical significance, a P-value of less than 0.05 was considered significant. Additionally, for clinical significance, the SMD value was classified into small (SMD = 0.2), medium (SMD = 0.5), and large (SMD = 0.8) effects [27]. Heterogeneity was assessed using Cochrane’s I^2^ test, with the following thresholds as per Cochrane recommendations: 0%–40% (might not be important), 30%–60% (may represent moderate heterogeneity), and 50%–100% (may represent substantial heterogeneity) [27]. In the evaluation of publication bias, MedCalc software version 22.017 (MedCalc Software Ltd., Belgium) was employed to conduct both Egger’s and Begg’s tests.

## Results

### Study selection

The study selection process, illustrated in Fig 1, followed a structured and rigorous approach to ensure the inclusion of the most relevant and high-quality research articles related to the effects of plantar sensory stimulation on lower limb motor function and gait in stroke patients.

Initially, a comprehensive search was conducted across multiple databases and registers to identify potential studies. A total of 14,447 records were identified from various databases including PubMed (9,962 records), Scopus (1,205 records), Web of Science (1,000 records), and Google Scholar (2,280 records). Additionally, an alternative search method identified 7 more records from website sources (5 records) and citation searches (2 records).

Before any formal screening, 4,281 duplicate records were identified and removed, resulting in 10,166 unique records to be screened. The preliminary screening involved title assessment, wherein 9,990 records were excluded as they were deemed irrelevant to the research question. This was followed by an abstract-level screening, which further eliminated 149 records. At this juncture, 27 records were deemed potentially relevant and were selected for a full-text review. Concurrently, from the alternative search method, all seven records were considered for full-text review.

During the in-depth full-text review, several exclusion criteria were applied. For the primary database search, 18 studies were excluded because they did not focus on plantar sensory stimulation. Two studies were ruled out due to the inclusion of placebo, control, or other interventions that did not align with the review’s focus. Finally, three studies were removed as they did not specifically address lower limb motor function and gait outcomes. Similarly, for the alternative search method, three studies were excluded for not being randomized control trials.

Following these rigorous exclusion steps, a total of eight studies, each fitting the eligibility criteria perfectly, were included in the systematic review and meta-analysis. These studies form the foundation upon which the review’s findings and conclusions are built.

### Risk of bias assessment

**Fig 2:**
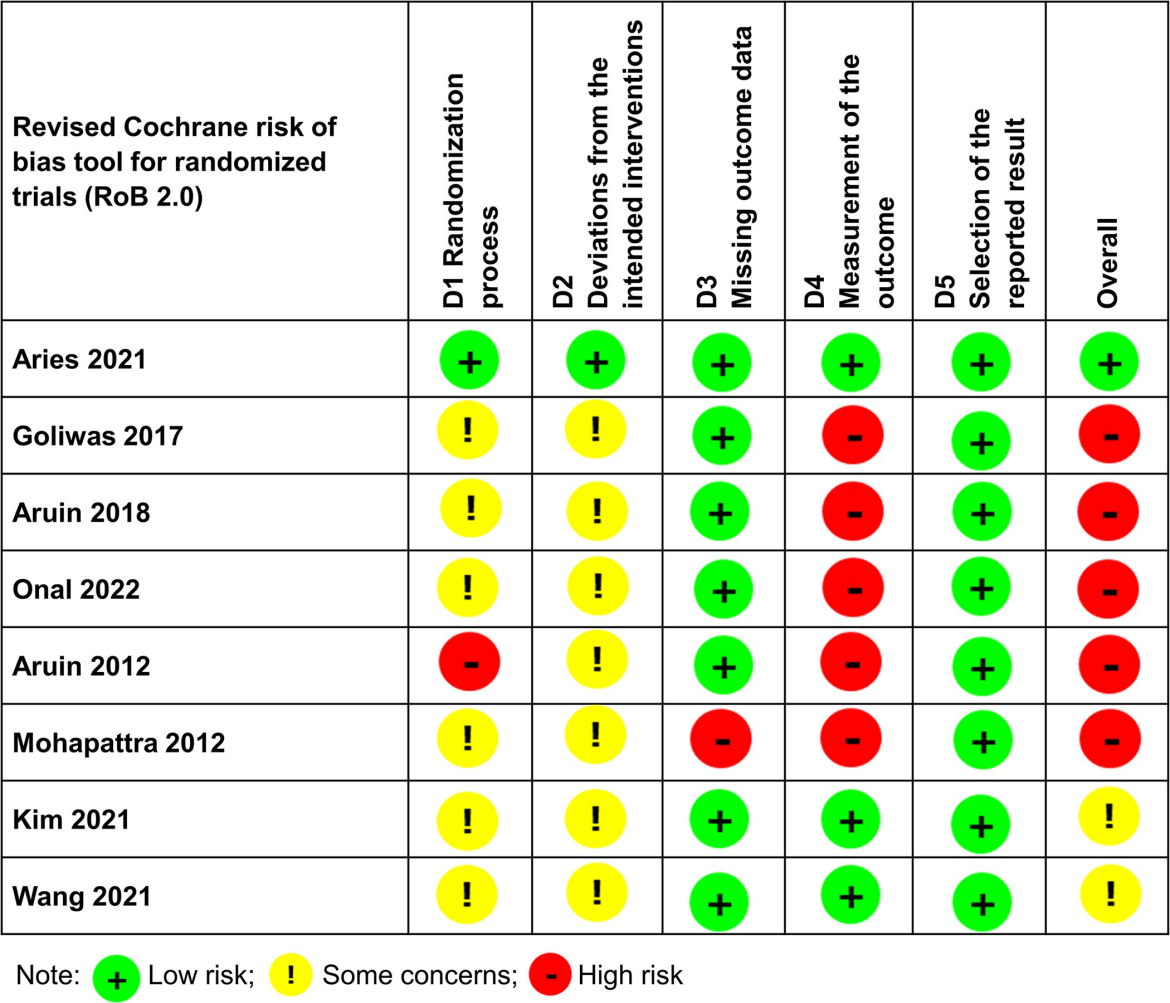
Risk of bias assessment using the revised Cochrane risk of bias tool for randomized trials (RoB 2.0). The figure summarizes the risk of bias across key domains, including randomization, blinding, and outcome reporting. Green indicates low risk, yellow represents some concerns, and red denotes high risk.

Our risk of bias assessment, employing the ROB2 tool and illustrated in Fig 2, yielded several key insights. Aries et al. (2021) emerged as the sole study with a consistent low risk across multiple categories [28]. In the realm of randomization processes, only Aries et al. (2021) was classified as low risk [28], while six other studies presented some concerns, and Aruin et al. (2012) was deemed high risk [29]. A notable high risk was identified in the deviations from intended interventions for seven studies, contrasting with Aries et al. (2021) which remained at low risk [28]. When evaluating bias due to missing outcome data, seven studies demonstrated a low risk, leaving Mohapatra et al. (2012) as the only high-risk entity [30]. In the assessment of outcome measurement bias, three studies were characterized as low risk, while the remaining five exhibited a high-risk profile. Impressively, all included studies uniformly presented a low risk of bias in the selection of reported results, underscoring a degree of reliability in this domain.

### Participants

This systematic review comprised a total of 215 participants. The mean time since stroke onset varied from 11 days [30] to 6 years [31]. Regarding stroke type, five studies included participants with both ischemic and hemorrhagic stroke [28, 30, 32–34]. One study exclusively enrolled participants with ischemic stroke [35], while two studies did not provide details about the stroke type [29, 31]. The summary of the included studies is presented in Table 1.

**Table 1:** Summary of included studies.

Study, design, sample size	Study group	N (SEX: M/F)	Side of paresis L/R	Age Mean (SD) (Years)	Time poststroke Mean (SD)	Type of stroke	Results of the somatosensory function
Aries 2021 [28], Pilot RCT, 34	Mobilization and Tactile Stimulation (MTS) + Task-Specific Gait Training (TSGT) *Duration per session*: 30–60 minutes for MTS, 30 minutes for TSGT *Frequency*: 5 times per week *Total duration*: 6 weeks	19 (9/10)	11/8	73.8 (14.1)	59.5 (18.1) days	CI 17; CH 2	**SWMs**: Mixed improvements in touch-pressure thresholds with some reductions at follow-up. No consistent trend across all sites. Proprioception or other subdomains not assessed.
	Textured Insoles (TI) + TSGT *Duration per session*: >30 minutes for TI, 30 minutes for TSGTT *Frequency*: 5 times per week *Total duration*: 6 weeks	15 (9/6)	9/6	72.4 (9.8)	53.9 (12.4) days	CI 12; CH 3	**SWMs**: Better retention or improvements at some sites, suggesting a beneficial effect of textured insoles on tactile sensory function. Proprioception not measured.
Goliwas 2017 [35], RCT, 37	Standard rehabilitation programe + Sensorimotor foot stimulation *Duration per session*: 30–45 minutes for standard rehabilitation + 20 minutes Sensorimotor foot stimulation *Frequency*: 5–6 days per week *Total duration*: 6 weeks	20 (12/8)	10/10	61.4 (8.0)	3.5 (2.3) years	CI 20; CH 0	**SWMs**: No significant improvement in touch-pressure thresholds. Other sensory subdomains not measured.
	standard rehabilitation programme *Duration per session*: 30–45 minutes for standard rehabilitation *Frequency*: 5–6 days per week *Total duration*: 6 weeks	17 (Not stated)	8/9	65.8 (9.2)	3.9 (2.5) years	CI 17; CH 0	**SWMs**: No significant changes in sensory thresholds. Other subdomains not assessed.
Aruin 2018 [31], RCT,10	Textured insole in shoe on the unaffected side + physical therapy *Duration per session*: 60 minutes *Frequency*: 3 days per week *Total duration*: 6 weeks	5 (Not stated))	1/4	59.33 (5.12)	6.28 (2.75) years	Not stated	Not measured: No direct assessment of somatosensory subdomains
	Physical therapy *Duration per session*: 60 minutes *Frequency*: 3 days per week *Total duration*: 6 weeks	5 (Not stated)	2/3			Not stated	Not measured: No direct assessment of somatosensory subdomains
Önal 2022 [32], RCT, 30	Plantar vibration + conventional physical therapy *Duration per session*: 15 minutes vibration + 45 minutes conventional physical therapy *Frequency*: 3 sessions with vibration, 2 with CPT only *Total duration*: 4 weeks	18 (9/6)	8/7	60 (9)	12 (3 to 24) months (median (IQR))	CI 10; CH 5	Not measured: No direct assessment of somatosensory subdomains
	Conventional physical therapy *Duration per session*: 60 minutes conventional physical therapy *Frequency*: 5 sessions per week *Total duration*: 4 weeks	15 (11/4)	5/10	59 (9)	14 (6 to 39) months (median (IQR))	CI 7; CH 8	Not measured: No direct assessment of somatosensory subdomains
Aruin 2012 [29], RCT, 18	Compelled Body Weight Shift (CBWS) therapy + physical therapy *Duration per session*: 60 minutes conventional physical therapy *Frequency*: 1 session + daily home exercises *Total duration*: 6 weeks	9 (Not stated)	4/5	57.7 (11.9)	6.7 (3.9) years	Not stated	Not measured: No direct assessment of somatosensory subdomains
	Physical therapy *Duration per session*: 60 minutes conventional physical therapy *Frequency*: 1 session + daily home exercises *Total duration*: 6 weeks	9 (Not stated)	4/5			Not stated	Not measured: No direct assessment of somatosensory subdomains
Mohapattra 2012 [30], RCT, 11	Compelled Body Weight Shift (CBWS) therapy + conventional physical therapy *Duration per session*: 90 minutes weekdays, 30 minutes on Saturdays *Frequency*: 6 sessions *Total duration*: 2 weeks	5 (3/2)	3/2	43.8 (4.7)	11 (0.9) days	CI 3; CH 2	Not measured: No direct assessment of somatosensory subdomains
	Conventional physical therapy Duration per session: 90 minutes weekdays, 30 minutes on Saturdays Frequency: 6 sessions Total duration: 2 weeks	6 (4/2)	3/3	53.7 (5.1)	18 (6) days	CI 2; CH 4	Not measured: No direct assessment of somatosensory subdomains
Kim 2021 [33], RCT, 25	Task-specific training (TST) + cognitive sensorimotor exercise (CSE) *Duration per session*: 60 minutes (30 minutes each for TST and CSE) *Frequency*: 5 sessions *Total duration*: 8 weeks	13 (7/9)	6/7	50.23 (14.89)	12.07 (3.57) months	CI 6; CH 7	**Proprioception:** Significant improvement in proprioception, with the mean proprioception error significantly reduced (mean change: -3.10 degrees) compared to the other groups.
	Conventional physical therapy *Duration per session*: 60 minutes *Frequency*: 5 sessions *Total duration*: 8 weeks	12 (8/4)	7/5	55.08 (10.55)	11.83 (3.71) months	CI 8; CH 4	**Proprioception:** Minimal change in proprioception (mean change: -0.58 degrees).
Wang 2021 [34], RCT, 50	Conventional gait training + customized insoles *Duration per session*: 40 minutes conventional gait training + 1 hour wear customized insoles *Frequency*: 5 sessions *Total duration*: 4 weeks	25 (19/6)	17/8	56.00 (49.50 to 66.50) years (median (IQR))	130.36 (64.87) days	CI 12; CH 13	Not measured: No direct assessment of somatosensory subdomains
	Conventional gait training *Duration per session*: 40 minutes conventional gait training *Frequency*: 5 sessions *Total duration*: 4 weeks	25 (18/7)	18/7	60.00 (54.00 to 65.00) years (median (IQR))	123.08 (54.06) days	CI 9; CH 16	Not measured: No direct assessment of somatosensory subdomains

Abbreviations: RCT: Randomized Controlled Trial; MTS: Mobilization and Tactile Stimulation; TI: Textured Insole; SWMs: Semmes Weinstein Monofilaments; CBWS: Compelled Body Weight Shift; TST: Task-Specific Training; CSE: Cognitive Sensorimotor Exercise; L: Left; R: Right; M: Male; F: Female; CI: Cerebral Infarct; CH: Cerebral Hemorrhage.

As shown in Table 1, improvements in somatosensory subdomains, such as touch-pressure thresholds, were observed [28, 33]. These findings suggest that interventions, such as textured insoles and cognitive sensorimotor exercises, may enhance specific aspects of sensory function. However, other studies did not report significant changes in somatosensory measures or did not assess these subdomains at all. The inconsistency in outcomes may be attributed to variations in the type, intensity, and duration of the interventions, or differences in baseline sensory impairments across participants.

### Intervention

Several different sensory stimulation techniques were employed in the included trials. Two trials utilized textured insoles as the sensory stimulation [28, 31]. Two studies used compelled body weight shift using uneven insoles [29, 30]. One study used customized insoles [34]. One study implemented sensorimotor foot stimulation [35], encompassing interventions such as improving flexibility of soft tissues around the ankle and foot, enhancing foot sensation through various tactile techniques, and promoting selective movements within the ankle joint using visual, auditory, and sensory stimuli. Additionally, this study focused on standing on surfaces with different textures. One study used task-specific training combined with cognitive sensorimotor exercise which consisted of proprioceptive training, tactile training, heel pressure, and spatial tasks [33]. Another study employed plantar vibration as the sensory stimulation [32]. Time for the intervention was from 20 min to 60 min, intervention ranged from three sessions per week [31] to six sessions per week [35]. Duration of intervention was from two weeks [30] to eight weeks [33].

Six included studies evaluated lower limb motor function using the following outcome measures: Modified Rivermead Mobility Index [28], Fugl-Meyer Lower Extremity Score [30, 31, 34], Fugl Meyer Assessment Scale [35], and Timed-Up and Go test [32]. The estimates of treatment effect and standardized mean difference for the studies measuring lower limb motor function are presented in Table 2. Additionally, seven of the studies assessed gait using the following outcome measures: 5-meter walk test [28], 10-meter walk test [32], and gait velocity [29–31, 33], and Tinetti Gait Scale [34]. The estimates of treatment effect and standardized mean difference for the studies measuring gait are shown in Table 3. Only one study of the included studies monitored adverse effects of pain and fatigue on the participants [28]. The psychometric properties of the outcome measures used in this review are summarized in Table 4. Notably, most measures demonstrated excellent reliability and validity, particularly the Fugl-Meyer Lower Extremity Score and the Modified Rivermead Mobility Index. However, there is a lack of data on the MCID for several measures, including the Timed-Up and Go test and the 5-Meter Walk Test, suggesting the need for further research to establish clinical benchmarks.

**Table 2:** Estimates of treatment effect and standardization mean difference on lower limb motor function.

Study	N	Outcome measure	Groups	Mean difference from baseline (SD)	Standardized mean difference [95%CI]
Aries 2021	34	Modified Rivermead Mobility Index (mRMI)	Experimental group (n = 15): task-specific gait training + unlimited textured insole wearing	7.75 (7.89)	0.71 [0.01, 1.41]
			Control group (n = 19): task specific gait training + mobilization and tactile stimulation	3.03 (5.12)	
Aruin 2018	10	Lower extremity Fugl–Meyer assessment (FMA-LE)	Experimental group (n = 5): physical therapy and textured insole	3.86 (3.44)	0.66 [-0.63, 1.96]
			Control group (n = 5): physical therapy	-0.50 (7.68)	
Goliwas 2017	37	Fugl Meyer Assessment Scale	Experimental group (n = 20): standard rehabilitation program and sensorimotor foot stimulation	5.70 (8.84)	0.38 [-0.27, 1.03]
			Control group (n = 17): standard rehabilitation program	2.30 (8.56)	
Önal 2022	30	Timed-Up and Go (s)	Experimental group (n = 15): plantar vibration therapy	-3.25 (0.99)	2.03 [1.13, 2.94]
			Control group (n = 15): conventional physical therapy	-1.50 (0.65)	
Mohapatra 2012	10	Lower extremity Fugl–Meyer assessment (FMA-LE)	Experimental group (n = 5): a two-week conventional physical therapy combined with Compelled Body Weight Shift (CBWS)	12 (0.86)	-0.37 [-1.58, 0.83]
			Control group (n = 5): a two-week conventional therapy	12.7 (2.17)	
Wang 2021	50	Lower extremity Fugl–Meyer assessment (FMA-LE)	Experimental group (n = 25): Conventional gait training + wear customized insoles for at least 1 h per day for four weeks	7 (1.86)	0.83 [0.24, 1.41]
			Control group (n = 25): Conventional gait training	5.48 (1.73)	

**Table 3:** Estimates of treatment effect and standardization mean difference on gait.

Study	N	Outcome measure		Mean difference from baseline (SD)	Standardized mean difference (95%CI)
Aries 2021	34	5-m walk test (s)	Experimental group (n = 15): task-specific gait training + unlimited textured insole wearing	-13.85 (22.70)	0.18 [-0.50, 0.86]
			Control group (n = 19): task specific gait training + mobilization and tactile stimulation	-9.96 (19.09)	
Aruin 2018	10	Gait velocity (cm/s)	Experimental group (n = 5): physical therapy and textured insole	4.93 (4.38)	0.64 [-0.65, 1.93]
			Control group (n = 5): physical therapy	-0.01 (8.95)	
Wang 2021	50	Tinetti Gait Scale (TGS)	Experimental group (n = 25): Conventional gait training + wear customized insoles for 1 h/day for 4 weeks	1.92 (2.01)	1.40 [0.44, 2.36]
			Control group (n = 25): Conventional gait training	1.12 (1.61)	
Aruin 2012	18	Gait velocity (m/s)	Experimental group (n = 9): a six-week physical therapy combined with Compelled Body Weight Shift (CBWS) therapy	0.06 (0.24)	0.17 [-0.76, 1.09]
			Control group (n = 9): only physical therapy	0.00 (0.40)	
Kim 2021	25	Gait velocity (m/s)	Experimental group (n = 13): Task-specific training combined with cognitive sensorimotor exercise	0.31 (0.08)	2.85 [1.69,4.02]
			Control group (n = 12): Conventional physical therapy training group	0.10 (0.06)	
Mohapatra 2012	10	Gait velocity (m/s)	Experimental group (n = 5): a two-week conventional physical therapy combined with Compelled Body Weight Shift (CBWS)	0.38 (0.09)	3.49 [1.32, 5.67]
			Control group (n = 5): a two-week conventional therapy	0.11 (0.05)	
Önal 2022	30	10-m walk test (s)	Experimental group (n = 15): plantar vibration therapy	-4.75 (1.48)	3.17 [2.05, 4.29]
			Control group (n = 15): conventional physical therapy	-1.00 (0.68)	

**Table 4:** Psychometric properties of outcome measures for lower limb motor function and gait in stroke patients.

Outcome Measure	Validity	Reliability	Responsiveness	MCID
Modified Rivermead Mobility Index (mRMI)	Good content, predictive, and convergent validity for stroke patients [36, 37].	Excellent test-retest (ICC 0.97–0.99), inter-rater (ICC 0.93), and intra-rater reliability (ICC 0.99) [36–38].	Highly responsive with a standardized response mean (SRM) of 1.31 [36].	The minimum detectable change (MDC) of 1.3 points [36].
Fugl-Meyer Lower Extremity Score (FMA-LE)	High construct and concurrent validity [39].	Excellent intra-rater and inter-rater reliability (ICC = 0.98) [39, 40].	Moderate to large effect sizes [41].	3.23 (inter-rater), 1.24 (intra-rater) [39].
Fugl Meyer Assessment Scale	High concurrent validity (r = 0.78–0.95) [41].	Excellent inter-rater (ICC = 0.93 to 0.96) and test-retest reliability (ICC = 0.88–0.98) [41, 42].	High sensitivity to change (SRM = 0.67–1.42) [41, 43].	Ranges from 4 to 13 points [42, 44].
Timed-Up and Go (TUG) test	Strong correlation with BBS and DGI validity (r = 0.97) [45].	Excellent test-retest and inter-rater reliability (ICC = 0.95–0.98) [45, 46].	Sensitive to changes in mobility, MDC = 3.2 seconds [45].	No data available
5-Meter Walk Test (5MWT)	No data available	No data available	High sensitivity to change (SRM = 1.22) [47].	No data available
10-Meter Walk Test (10MWT)	Strong construct and concurrent validity with other walking and balance tests [48].	Excellent test-retest and inter-rater reliability (ICCs: 0.76–0.99) [48, 49].	Sensitive to changes in walking ability, suitable for tracking rehabilitation progress [50].	MCID = 0.16 m/s [51].
Gait Velocity	Good convergent validity with the modified Emory Functional Ambulation Profile (mEFAP) and the Rivermead Mobility Index, with correlation coefficients ≥0.67 [52].	Excellent test-retest reliability (ICC = 0.997) [52].	Changes in gait velocity are significantly correlated with improvements in Functional Ambulatory Category (FAC) [53].	MCID = 0.18–0.25 m/s (anchor-based); 0.13–0.15 m/s (distribution-based) [54].
Tinetti Gait Scale (TGS)	Excellent convergent validity with other clinical assessments such as the Trunk Impairment Scale (TIS), Postural Assessment Scale for Stroke (PASS), and Functional Ambulatory Categories (FAC) in subacute stroke [55].	High test-retest reliability (ICC = 0.84) [56]	Moderate correlations with changes in functional measures [56].	MCID = 1 points [57].

### Effect of plantar sensory stimulation on lower limb motor function

**Fig 3:**
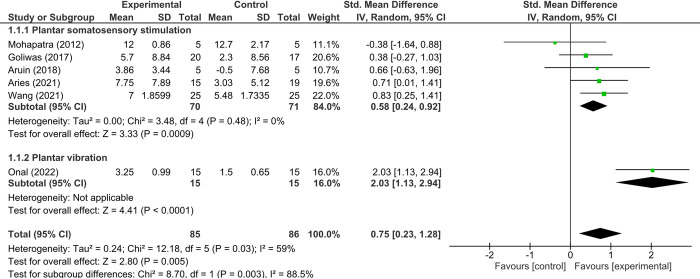
Forest plot of effect of sensory stimulation on lower limb motor function.

The forest plot in Fig 3 illustrates the effect of plantar sensory stimulation on lower limb motor function, showing a medium effect size (SMD = 0.75 [95% CI = 0.23–1.28]; P = 0.005; I^2^ = 59%), indicating moderate heterogeneity across studies. To explore the sources of this heterogeneity, subgroup analyses were conducted based on the type of sensory intervention. The analyses revealed that specific types of plantar somatosensory stimulation, such as tactile stimulation or textured insoles, demonstrated a more consistent effect, with a medium effect size (SMD = 0.58 [95% CI = 0.24–0.92]; P = 0.0009; I^2^ = 0%), suggesting that the more uniform application of these interventions helped to reduce variability. In contrast, studies using plantar vibration produced a large effect size (SMD = 2.03 [95% CI = 1.13–2.94]; P < 0.0001), but heterogeneity was not applicable due to data from a single trial. This indicates that variations in intervention type and application protocols contributed to the overall heterogeneity observed. The analysis revealed an absence of significant publication bias for the lower limb motor function, as evidenced by the results of Egger’s test (intercept = -0.53, 95% CI = -0.871 to 7.65, *P* = 0.87) and Begg’s test (Kendall’s Tau = -0.20, *P* = 0.57).

### Effect of plantar sensory stimulation on gait

**Fig 4:**
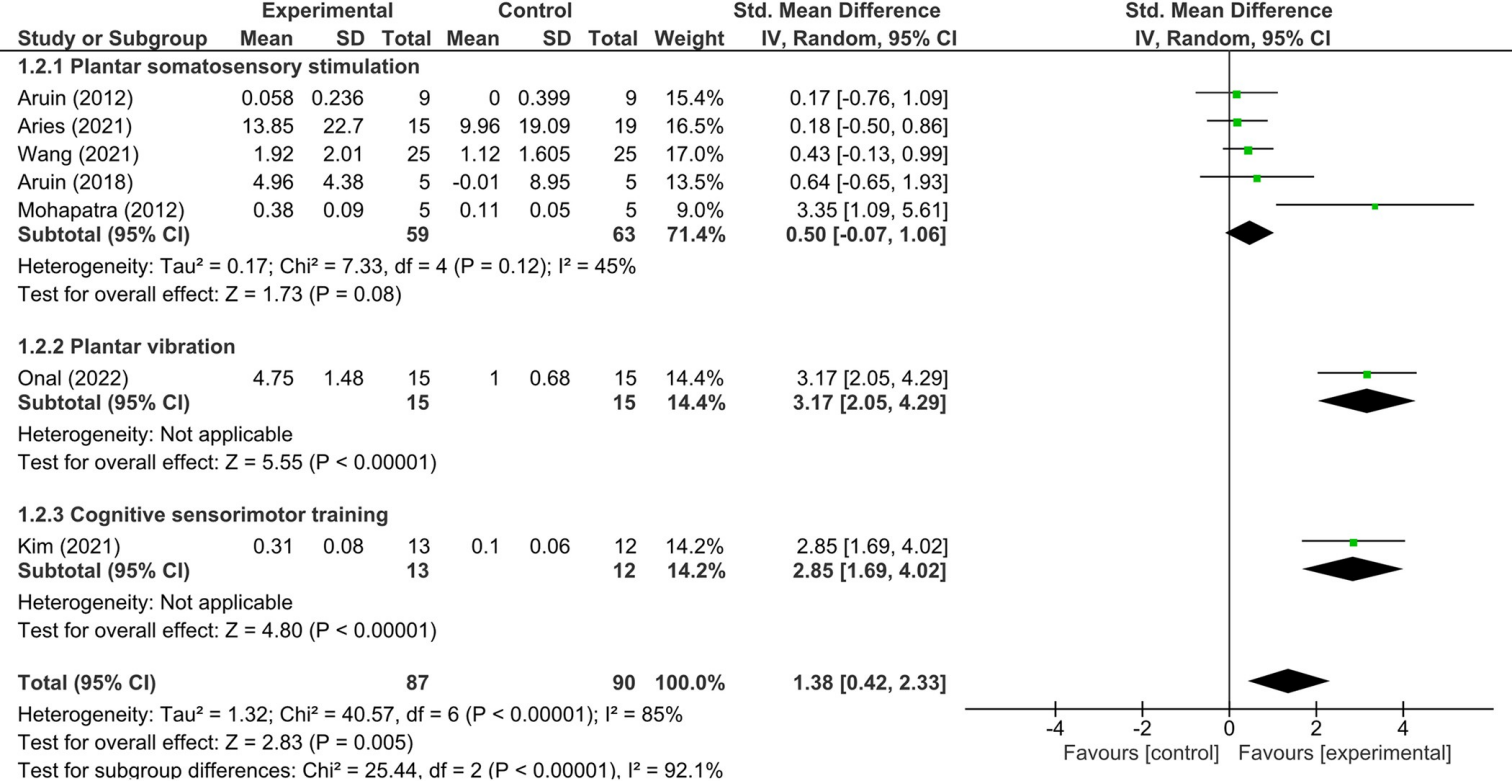
Forest plot of effect of sensorimotor stimulation on gait.

For gait outcomes, the forest plot in Fig 4 shows a large overall effect size (SMD = 1.38 [95% CI = 0.42–2.33]; P = 0.005; I^2^ = 85%), reflecting high heterogeneity. Subgroup analyses based on the type of sensory intervention provided further insights. While interventions like plantar somatosensory stimulation delivered via textured insoles or tactile methods showed moderate and varied results (SMD = 0.50 [95% CI = -0.07–1.06]; P = 0.08; I^2^ = 45%), other approaches such as plantar vibration and cognitive sensorimotor training demonstrated large effect sizes (SMD = 3.17 [95% CI = 2.05–4.29]; SMD = 2.85 [95% CI = 1.69–4.02], respectively) with lower variability within their subgroups (I^2^ not applicable for single trials). These findings suggest that the type, intensity, and consistency of interventions, along with patient-specific factors, played a role in the heterogeneity observed across studies. Subsequently, the analysis revealed an absence of significant publication bias for the gait, as evidenced by the results of Egger’s test (intercept = 5.4, 95% CI = -0.73 to 11.59, *P* = 0.07) and Begg’s test (Kendall’s Tau = 0.43, *P* = 0.18).

## Discussion

Stroke stands as one of the foremost causes of long-term disability worldwide, profoundly affecting the adult population and presenting a global health challenge [58, 59]. The rehabilitation journey post-stroke is multifaceted, with paramount emphasis on reviving voluntary activity in the hemiparetic lower extremity and recuperating the ability to walk autonomously [31, 60, 61]. Such recovery is instrumental in enabling stroke survivors to reclaim their independence, reintegrate into societal settings, and enjoy a quality of life akin to their pre-stroke state. Yet, while the quest for effective rehabilitation methods continues, a pronounced lacuna exists regarding the potential of plantar sensory stimulation as a rehabilitative intervention for lower limb motor function and gait among stroke patients. Our research ambitiously sought to fill this knowledge chasm through an exhaustive systematic review and meta-analysis of the available literature.

Emerging from our investigation is the revelation that plantar sensory stimulation offers a promising avenue for enhancing lower limb motor functionality, as evidenced by a medium effect size (SMD = 0.75). Delving deeper into the modalities, the subgroup analysis shone light on specific techniques, particularly the use of textured insoles or tactile stimulations, that exhibited tangible improvements, indicated by a medium effect size (SMD = 0.58). Furthermore, the efficacy of plantar vibration was unmistakably underscored, showcasing a large effect size (SMD = 2.03). Such data not only provides empirical support for plantar sensory stimulation’s potential but also offers insights into the intricate neurophysiological processes possibly at work. As elucidated by Bonassi et al. (2017), the underpinnings of these improvements may lie in the realm of neuroplasticity, with sensory-based interventions acting as catalysts for adaptive motor cortical changes—a potent mechanism facilitating post-stroke recovery [62].

The interpretation of these findings is strengthened using outcome measures with robust psychometric properties. In our review, measures such as the Fugl-Meyer Lower Extremity Score and the Modified Rivermead Mobility Index exhibited excellent reliability and known MCID values, supporting the clinical relevance of the improvements observed. However, it is important to note that certain measures, like the Timed-Up and Go test and 5-Meter Walk Test, lacked comprehensive data on MCID, which limits our ability to fully assess the clinical significance of changes. Future studies should aim to establish these psychometric benchmarks to improve the consistency and interpretability of rehabilitation outcomes in this field.

Our analysis of lower limb motor function and gait outcomes demonstrated varying levels of effectiveness across different interventions. For lower limb motor function, studies like Wang (2021) showed significant improvements on the Fugl-Meyer Lower Extremity Assessment, with a mean change of 7 (1.86) and an SMD of 0.83 [34], surpassing known MDC thresholds of 1.3 [36], indicating reliable and clinically meaningful gains. Similarly, the Modified Rivermead Mobility Index (mRMI) in Aries (2021) showed moderate improvement with an SMD of 0.71 [28]. In contrast, Mohapatra (2012) displayed limited effectiveness with negative effect sizes [30], suggesting variability in response to certain interventions. For gait outcomes, the 10-Meter Walk Test in Önal (2022) reported a mean improvement of -4.75 (1.48) seconds with an SMD of 3.17 [32], indicating substantial gains in walking speed. Furthermore, gait velocity improvements seen in Kim (2021) and Mohapatra (2012) exceeded the MCID of 0.18–0.25 m/s [54], underscoring the clinical relevance of these changes. These findings suggest that interventions combining sensory stimulation with motor training can lead to robust outcomes in lower limb rehabilitation.

Parallel to these findings, our study also highlights the instrumental role of sensory input in bolstering stroke recovery [63]. Historically, the emphasis in stroke rehabilitation has often veered more towards motor function, perhaps overlooking the symbiotic relationship between sensory and motor recovery. While the significance of sensory feedback in shaping motor responses is increasingly recognized, its full potential remains largely untapped in clinical rehabilitation [12, 64]. Consequently, our findings serve as a clarion call for both deeper research endeavors into sensory stimulation techniques and their subsequent infusion into mainstream rehabilitative practices.

In assessing gait improvements, our findings were compelling. Plantar sensory stimulation emerged as a formidable intervention, eliciting substantial enhancements in gait metrics with a large effect size (SMD = 1.38). Further granularity provided by the subgroup analyses reaffirmed the benefits of both plantar vibration and cognitive sensorimotor training. The implications of these findings are vast, suggesting not only the merit of sensory interventions but also hinting at the potential neurological pathways they may engage.

The variability observed in effect sizes and mean differences across studies could be attributed to several factors, including differences in intervention protocols, intensity, and participant characteristics. Interventions that combined physical therapy with sensory inputs, such as textured insoles or vibration therapy, generally produced stronger outcomes. This indicates a synergistic effect that might not be present with standard rehabilitation alone.

Plantar somatosensory stimulation, such as the use of textured insoles, primarily targets foot sensory receptors, enhancing feedback to the central nervous system, which may contribute to improved motor function and balance. This mechanism is likely to drive motor recovery by fostering sensory-motor integration, providing continuous low-level input that aids in maintaining balance and stability [62]. However, the effects observed with textured insoles were moderate, possibly due to the passive nature of the stimulation, which may not always provide sufficient intensity to produce significant changes across all motor functions. In contrast, plantar vibration involves sub-threshold stimulation that activates proprioceptive receptors more directly, leading to an increase in sensory input [65]. This more intensive, direct engagement of sensory pathways might explain the larger effect sizes observed in gait improvements, as the vibration enhances proprioceptive feedback and sensory-motor integration specific to gait. Additionally, vibratory stimulation has been linked to reduced variability in toe clearance during walking, leading to more consistent and stable gait patterns [66]. The targeted, dynamic nature of vibratory feedback may therefore offer a stronger influence on gait-related outcomes compared to the more general stimulation provided by textured insoles.

Our risk of bias assessment, conducted using the ROB2 tool, revealed varying levels of methodological quality across the included studies. While Aries et al. (2021) demonstrated a consistently low risk across multiple domains, other studies presented concerns [28]. For instance, six studies had issues with the randomization process, and Aruin et al. (2012) was deemed high risk in this domain [29]. Additionally, seven studies showed a high risk of deviations from intended interventions, which might have impacted adherence to protocols. Outcome measurement bias was notably high in five studies, indicating possible inconsistencies in how outcomes were assessed and reported. However, all studies maintained a low risk of bias regarding the selection of reported results, which suggests reliable reporting. These varying levels of bias may affect the generalizability of the findings, emphasizing the need for future studies to improve on aspects such as randomization, blinding, and intervention consistency.

The observed improvements in somatosensory function, particularly in tactile sensation, suggest that specific interventions such as textured insoles, cognitive sensorimotor exercises, and somatosensory stimulation can effectively enhance sensory feedback in stroke survivors. Electrical stimulation applied to the paretic foot significantly improved precision in foot placement, especially among individuals with greater impairment, indicating that targeted sensory inputs can enhance motor control [67]. Similarly, retraining leg somatosensory function led to significant improvements in balance, although gait enhancements were not as pronounced, suggesting that while sensory retraining can improve stability, more refined approaches may be needed to impact gait directly [68]. Integrating somatosensory training with standard physical therapy also resulted in better neuromuscular control, as evidenced by improvements in Functional Independent Measure and Electroencephalography scores, highlighting the role of sensory retraining in supporting functional and neural recovery [69]. These findings align with the idea that stimulating sensory receptors through continuous or targeted inputs can help re-establish neural pathways disrupted by stroke.

However, the variability in the effectiveness of these interventions across studies indicates that factors such as the type of sensory impairment, the intensity of interventions, or the specific sensory subdomains targeted may influence outcomes. Future research should explore whether certain aspects of sensory function, like proprioception, require more intensive or prolonged stimulation to achieve significant improvements and how these interventions can be optimized to enhance both balance and gait recovery.

This study, though illuminating, has its limitations. The comprehensive search strategy excluded non-English publications and non-randomized controlled trials. The meta-analysis was grounded in a limited set of randomized controlled trials, which may have impacted statistical potency. Additionally, the absence of established psychometric benchmarks such as MCID for certain outcome measures, including the Timed-Up and Go test and 5-Meter Walk Test, limits the ability to fully interpret the clinical relevance of the observed changes. Future research should focus on standardizing these measures and establishing robust psychometric data to allow for more consistent comparisons across studies. Inconsistencies in study protocols and outcome measures across investigations contributed to identified statistical heterogeneity. With some subgroups anchored by just one study, interpretation becomes complex. Therefore, future studies should strive to unify outcome measurements for lower limb motor function and gait to allow for more robust comparisons and draw firmer conclusions.

## Conclusion

Our systematic review and meta-analysis revealed a noteworthy positive influence of plantar sensory stimulation on both lower limb motor function and gait in stroke patients. Various techniques, such as plantar somatosensory stimulation, plantar vibration, and cognitive sensorimotor training, demonstrated significant enhancement in motor function and gait outcomes compared to control groups. Notably, the use of plantar somatosensory stimulation, particularly employing textured insoles or tactile methods, and plantar vibration yielded medium to large effect sizes, indicating their potential efficacy in rehabilitation for stroke patients. This review underscores the importance of plantar sensory stimulation techniques in post-stroke recovery, emphasizing the need for more extensive and randomized controlled trials to further elucidate the optimal techniques and parameters for maximizing therapeutic outcomes.

Future research should explore the benefits of combining sensory stimulation with motor training exercises, as integrating both modalities could enhance motor learning and overall recovery. Additionally, incorporating new technologies, such as wearable sensors and robotic devices, may provide real-time feedback and adaptive stimulation, leading to more precise and effective interventions. To improve the comparability and reproducibility of results, standardizing intervention protocols—such as the duration, intensity, and frequency of sensory stimulation—across studies will be essential. Advancing these areas will help establish more effective rehabilitation strategies and maximize motor recovery outcomes for individuals with stroke.

## Supporting information

S1 Checklist PRISMA checklist. (DOCX)

S1 Table Search term of studies published from 1978 to September 2023, utilizing reputable and scholarly databases. (DOCX)

S2 Table Studies identified and excluded with reasons. (DOCX)
