# Supplementary material for: Plantar sensory stimulation and its impact on gait and lower limb motor function in individuals with stroke: A systematic review and meta-analysis
Source: PLoS One. 2024 Dec 6;19(12):e0315097. doi: 10.1371/journal.pone.0315097 (PMC11623553; doi:10.1371/journal.pone.0315097)
Supplement: S1 Table — (DOCX) [file pone.0315097.s002.docx]

**Supplemental Table 1. Search Term of studies published from 1978 to September 2023, utilizing reputable and scholarly databases.**

| Scopus  ALL ("stroke" OR "cerebrovascular accident" OR cva OR "cerebral stroke" OR "cerebrovascular stroke" AND "sensory stimulation" OR "plantar sensation" OR "foot sensation" OR "somatosensory training" OR "plantar pressure" OR "plantar vibration" AND placebo OR sham OR control AND "lower limb motor function" OR "lower extremity motor function" OR gait OR walking ) AND ( LIMIT-TO ( DOCTYPE , "ar" ) ) |
| --- |
| Web of Science  "stroke" OR "cerebrovascular accident" OR cva OR "cerebral stroke" OR "cerebrovascular stroke" AND "sensory stimulation" OR "plantar sensation" OR "foot sensation" OR "somatosensory training" OR "plantar pressure" OR "plantar vibration" AND placebo OR sham OR control AND "lower limb motor function" OR "lower extremity motor function" OR gait OR walking |
| PubMed  ((((((((((((((((((stroke) OR ("cerebrovascular accident")) OR (CVA)) OR ("cerebral stroke")) OR ("cerebrovascular stroke")) AND ("sensory stimulation")) OR ("plantar sensation")) OR ("foot sensation")) OR ("somatosensory training")) OR ("plantar pressure")) OR ("plantar vibration")) AND (placebo)) OR (sham)) OR (control)) AND ("lower limb" "motor function")) OR ("lower extremity" "motor function")) OR (gait)) OR (walking)) |
| Google Scholar |
| "stroke" OR "cerebrovascular accident" OR cva OR "cerebral stroke" OR "cerebrovascular stroke" AND "sensory stimulation" OR "plantar sensation" OR "foot sensation" OR "somatosensory training" OR "plantar pressure" OR "plantar vibration" AND placebo OR sham OR control AND "lower limb motor function" OR "lower extremity motor function" OR gait OR walking |
