# Supplementary material for: Plantar sensory stimulation and its impact on gait and lower limb motor function in individuals with stroke: A systematic review and meta-analysis
Source: PLoS One. 2024 Dec 6;19(12):e0315097. doi: 10.1371/journal.pone.0315097 (PMC11623553; doi:10.1371/journal.pone.0315097)
Supplement: S2 Table — (DOCX) [file pone.0315097.s003.docx]

**Supplemental Table 2. Studies Identified and Excluded with Reasons**

| # | Title | Author | Journal | Year | Reason for exclusion |
| --- | --- | --- | --- | --- | --- |
| 61 | Effects of augmented somatosensory input using vibratory insoles to improve walking in individuals with chronic post-stroke hemiparesis | Liang, J.N.; Ho, K.-Y.; Hung, V.; Reilly, A.; Wood, R.; Yuskov, N.; Lee, Y.-J. | Gait and Posture | 2021 | Not Randomised Controlled Trials |
| 104 | Retrospective study of effect of whole-body vibration training on balance and walking function in stroke patients | Xie, L.; Yi, S.-X.; Peng, Q.-F.; Liu, P.; Jiang, H. | World Journal of Clinical Cases | 2021 | No Plantar sensory stimulation |
| 471 | A clinical single blind study to investigate the immediate effects of plantar vibration on balance in patients after stroke | Karimi-AhmadAbadi, A.; Naghdi, S.; Ansari, N.N.; Fakhari, Z.; Khalifeloo, M. | Journal of Bodywork and Movement Therapies | 2018 | Not Lower limb motor function and gait |
| 685 | Effects of vibratory training on plantar impression in patients affected by stroke | Silva, A.T.; Carvalho, A.J.B.; Andrades, M.F.; Calixto Junior, R.; Dias, M.P.F.; Silva, A.M.; Martinez, B.B.; Honorato, D.C. | International Journal of Therapy and Rehabilitation | 2016 | No Plantar sensory stimulation |
| 819 | Combining afferent stimulation and mirror therapy for rehabilitating motor function, motor control, ambulation, and daily functions after stroke | Lin, K.-C.; Huang, P.-C.; Chen, Y.-T.; Wu, C.-Y.; Huang, W.-L. | Neurorehabilitation and Neural Repair | 2014 | No Plantar sensory stimulation |
| 2700 | Standard early rehabilitation and lower limb transcutaneous nerve or neuromuscular electrical stimulation in acute stroke patients: a randomized controlled pilot study. | Yen, Hsiao-Ching; Chen, Wen-Shiang; Jeng, Jiann-Shing; Luh, Jer-Junn; Lee, Ya-Yun; Pan, Guan-Shuo | Clinical rehabilitation | 2019 | No Plantar sensory stimulation |
| 2736 | Intensive gait training with rhythmic auditory stimulation in individuals with chronic hemiparetic stroke: a pilot randomized controlled study. | Cha, Yuri; Kim, Young; Hwang, Sujin; Chung, Yijung | NeuroRehabilitation | 2014 | No Plantar sensory stimulation |
| 4038 | Rehabilitation of balance after stroke with multisensorial training: a single-blind randomized controlled study. | Yelnik, Alain P.; Le Breton, Frederique; Colle, Florence M.; Bonan, Isabelle V.; Hugeron, Caroline; Egal, Véronique; Lebomin, Elizabeth; Regnaux, Jean-Philippe; Pérennou, Dominic; Vicaut, Eric | Neurorehabilitation and neural repair | 2008 | No Plantar sensory stimulation |
| 5312 | Effects of different vibration frequencies on muscle strength, bone turnover and walking endurance in chronic stroke. | Yang, Zhenhui; Miller, Tiev; Xiang, Zou; Pang, Marco Y. C. | Scientific reports | 2021 | No Plantar sensory stimulation |
| 6727 | Bilateral Transcutaneous Electrical Nerve Stimulation Improves Lower-Limb Motor Function in Subjects With Chronic Stroke: A Randomized Controlled Trial. | Kwong, Patrick W. H.; Ng, Gabriel Y. F.; Chung, Raymond C. K.; Ng, Shamay S. M. | Journal of the American Heart Association | 2018 | No Plantar sensory stimulation |
| 6946 | No specific effect of whole-body vibration training in chronic stroke: a double-blind randomized controlled study. | Brogårdh, Christina; Flansbjer, Ulla-Britt; Lexell, Jan | Archives of physical medicine and rehabilitation | 2012 | No Plantar sensory stimulation |
| 6954 | Transcutaneous electrical nerve stimulation combined with task-related training improves lower limb functions in subjects with chronic stroke. | Ng, Shamay S. M.; Hui-Chan, Christina W. Y. | Stroke | 2007 | No Plantar sensory stimulation |
| 11170 | Changes in gait and plantar foot loading upon using vibrotactile wearable biofeedback system in patients with stroke | Ma, CZH; Zheng, YP; Lee, WCC | TOPICS IN STROKE REHABILITATION | 2018 | Not Randomised Controlled Trials |
| 11212 | The effect of external cues with vibratory stimulation on spatiotemporal gait parameters in chronic stroke patients | Park, JM; Lim, HS; Song, CH | JOURNAL OF PHYSICAL THERAPY SCIENCE | 2015 | No Plantar sensory stimulation |
| 11214 | Impact of Somatosensory Training on Neural and Functional Recovery of Lower Extremity in Patients with Chronic Stroke: A Single Blind Controlled Randomized Trial | Alwhaibi, RM; Mahmoud, NF; Basheer, MA; Zakaria, HM; Elzanaty, MY; Ragab, WM; Al Awaji, NN; Elserougy, HR | INTERNATIONAL JOURNAL OF ENVIRONMENTAL RESEARCH AND PUBLIC HEALTH | 2021 | Not Lower limb motor function and gait |
| 11651 | Long-term effects of 6-week whole-body vibration on balance recovery and activities of daily living in the postacute phase of stroke - A randomized, controlled trial | van Nes, IJW; Latour, H; Schils, F; Meijer, R; van Kuijk, A; Geurts, ACH | STROKE | 2006 | No Plantar sensory stimulation |
| 12300 | Effects of proprioception training with exercise imagery on balance ability of stroke patients | Lee, H; Kim, H; Ahn, M; You, Y | JOURNAL OF PHYSICAL THERAPY SCIENCE | 2015 | No Plantar sensory stimulation |
| 12319 | Effects of sensorimotor foot training on the symmetry of weight distribution on the lower extremities of patients in the chronic phase after stroke | Goliwas, M; Kocur, P; Furmaniuk, L; Majchrzycki, M; Wiernicka, M; Lewandowski, J | JOURNAL OF PHYSICAL THERAPY SCIENCE | 2015 | Not Lower limb motor function and gait |
| 12843 | Effects of Intensive Whole-Body Vibration Training on Muscle Strength and Balance in Adults With Chronic Stroke: A Randomized Controlled Pilot Study | Tankisheva, E; Bogaerts, A; Boonen, S; Feys, H; Verschueren, S | ARCHIVES OF PHYSICAL MEDICINE AND REHABILITATION | 2014 | No Plantar sensory stimulation |
| 12854 | Does whole-body vibration training in the horizontal direction have effects on motor function and balance of chronic stroke survivors? A preliminary study | Lee, G | JOURNAL OF PHYSICAL THERAPY SCIENCE | 2015 | No Plantar sensory stimulation |
| 12903 | The effects of whole-body vibration therapy on bone turnover, muscle strength, motor function, and spasticity in chronic stroke: a randomized controlled trial | Pang, MYC; Lau, RWK; Yip, SP | EUROPEAN JOURNAL OF PHYSICAL AND REHABILITATION MEDICINE | 2013 | No Plantar sensory stimulation |
| 14290 | The effects of High Frequency Transcutaneous Nerve Stimulation on Spasticity, Proprioception, Strength, and Balance in Patients with Stroke : A Randomized Controlled Trial | Lee, SA; Cha, HG | JOURNAL OF MAGNETICS | 2019 | No Plantar sensory stimulation |
| 15121 | The effect of swiss ball therapy on sit-to-stand function, paretic limb weight bearing and lower limb motor score in patients with hemiplegia | Rasheeda, V; Sivakumar, R | INTERNATIONAL JOURNAL OF PHYSIOTHERAPY | 2017 | No Plantar sensory stimulation |
